# Aberrant Hypermethylation of SALL3 with HPV Involvement Contributes to the Carcinogenesis of Cervical Cancer

**DOI:** 10.1371/journal.pone.0145700

**Published:** 2015-12-23

**Authors:** Xing Wei, Shaohua Zhang, Di Cao, Minyi Zhao, Qian Zhang, Juan Zhao, Ting Yang, Meili Pei, Li Wang, Yang Li, Xiaofeng Yang

**Affiliations:** 1 Department of Gynecology and Obstetrics, The First Affiliated Hospital of Medical School, Xi’an Jiaotong University, 277 West Yanta Road, Xi’an 710061, China; 2 Department of Gynecology and Obstetrics, The Affiliated Hospital of Xi’an Medical College, Xi’an 710077, China; 3 Center of Maternal and Child Health Care, The First Affiliated Hospital of Medical School, Xi’an Jiaotong University, 277 West Yanta Road, Xi’an 710061, China; Georgetown University, UNITED STATES

## Abstract

**Objective:**

This study aimed to investigate the methylation status of the promoter region of spalt-like transcription factor 3 (*SALL3*) and the expression of *SALL3* in cervical cancer to explore the function of this gene in cervical cancer carcinogenesis.

**Methods:**

The methylation status of *SALL3* was detected by methylation-specific PCR, and *SALL3* gene expression was assessed by real-time quantitative PCR in the cervical cancer cell lines, SiHa, HeLa and C33A, as well as in cervical cancer tissue samples (n = 23), matched pericarcinomatous tissue samples (n = 23) and normal cervix tissue samples (n = 17). MTT was used to measure the cell viability and proliferation capacity of SiHa and HeLa cells.

**Results:**

The *SALL3* promoter was completely methylated in SiHa cells, unmethylated in C33A cells and partially methylated in HeLa cells. After treatment of SiHa and HeLa cells with 5 μM and 10 μM of 5-Azacytidine (5-Aza), respectively, the methylation level of the *SALL3* promoter decreased and observed increase in the degree of unmethylation in a dose-dependent manner. Moreover, the relative expression of *SALL3* mRNA increased as the concentration of 5-Aza increased in SiHa (*p*<0.05) and HeLa (*p*<0.05) cells. This above-mentioned increase in *SALL3* mRNA in SiHa cells was more remarkable than that observed in HeLa cells. Cell proliferation capacity also decreased after administration of 5-Aza to SiHa and HeLa cells (*p*<0.05). Methylation of the *SALL3* promoter was observed in 15 of 23 (65.21%) cervical cancer tissue samples, 15 of 23 (65.21%) matched pericarcinomatous tissue samples and 5 of 17 (29.41%) normal cervical tissue samples (*p*<0.05). *SALL3* mRNA expression was significantly lower in cervical cancer and pericarcinomatous tissues compared with normal cervical tissues (*p*<0.05). In all cervix tissue samples, HPV infection was positively associated with hypermethylation of the promoter region of *SALL3* (*p*<0.05, r = 0.408), and the expression of *SALL3* mRNA in HPV-positive tissues was lower than that in HPV-negative tissues (*p*<0.05).

**Conclusion:**

The aberrant hypermethylation of *SALL3* together with HPV involvement inactivated its function as a tumor suppressor and contributed to carcinogenesis in cervical cancer.

## Introduction

Cervical cancer ranks as the second leading cause of genital tract cancer-related mortality among women worldwide, and results in approximately 266,000 deaths each year according to the recent National Comprehensive Cancer Network (NCCN) Guidelines (2015 version)[1].Over 99% of cases are linked to infection of the genitals with human papillomaviruses (HPVs), which have been identified in the etiology of cervical cancer and have infected approximately 660 million people[2]; however, the prevalence of HPV infection is still insufficient to account for all instances of cervical carcinogenesis. In the past few years, epigenetic mechanisms, especially DNA methylation, have emerged and have subsequently provided new insights into the occurrence of tumors[3, 4]. Nevertheless, scientists continue to explore the mechanisms of carcinogenesis and the development of cancers in genomics.

Spalt-like transcription factor 3 (*SALL3*) is a member of the *SAL* family and is located on 18q23. *SALL3* encodes a sal-like C_2_H_2_-type zinc-finger protein. Mutations in some of these genes are associated with congenital disorders in humans, which indicates their importance in embryonic development[5–7]. Shikauchi *et al*[8]found that *SALL3* was silenced by DNA methylation and that the protein product of this gene (SALL3) directly interacts with the PWWP domain of DNMT3A in human hepatocellular carcinoma (HCC), which suggested that *SALL3* acts as a tumor suppressor in HCC. Love *et al*[9] sequenced the exomes of 59 Burkitt lymphoma tumors, and the results showed for the first time that *SALL3* was recurrently mutated in Burkitt lymphomas. Furthermore, in CD133 (+) colorectal cancer cells (CRC), *SALL3* was significantly up-regulated compared with CD133 (-) CRC cells[10]. Moreover, a recent study utilizing high-throughput DNA methylation analysis also showed that *SALL3* was a potential biomarker for colon cancer[11]. The above-mentioned findings demonstrate that abnormal expression of *SALL3* is associated with the occurrence of several cancers. However, whether *SALL3* is involved in the carcinogenesis of cervical cancer remains unknown.

In our current study, we demonstrated that the promoter region of *SALL3* is hypermethylated and, together with HPV infection, is involved in cervical cancer; additionally, the expression level of SALL3 mRNA was down-regulated. We suggest that DNA methylation of *SALL3* inhibits its role as a tumor suppressor and contributes to the carcinogenesis of cervical cancer.

## Materials and Methods

### Ethics statement

This study was approved by the “Ethics Committee of the First Affiliated Hospital of Xi’an Jiaotong University” in Shannxi, China. Written informed consent was obtained from all patients for participation in this study.

### Cell lines and culture conditions

The three human cervical cancer cell lines SiHa、HeLa and C33A were given to us by Dr. Jing Ji from the First Affiliated Hospital of Medical School, Xi’an Jiaotong University[12, 13]. All these cells were cultured in the high glucose Dulbecco's Modified Eagle’s Medium (DMEM)(HyClone, USA) supplemented with 10% fetal bovine serum (FBS)(Si Ji Qing, China) at 37°C in an atmosphere of 5% CO_2_.

### 5-Azacytidine Treatment

1.0×10^5^/ well SiHa and HeLa cells were cultured separately in 6-well plates in DMEM with 10% FBS, and after 24 hours, the medium was replaced with fresh medium containing 0, 5 or 10 μM 5-Azacytidine (5-Aza)(Sigma, USA). The medium containing 5-Aza was replaced every 24 hours during a 72-hour period.

### MTT Assay

3×10^3^/ well SiHa or HeLa cells were seeded into 96-well plates and were cultured with 0, 5, or 10 μM 5-Aza. Throughout a 5-day period, the medium was replaced every 24 h, and the same concentration of 5-Aza was added. A 3-(4, 5-dimethylthiazol-3-yl)-2, 5-diphenyl tetrazolium bromide (MTT) assay was used to assess the proliferative capacity of SiHa and HeLa cells after treatment with 5-Azacytidine according to a standard protocol. Cell viability was measured by the absorbance at 490 nm.

### Patients and samples

Twenty-three cervical cancer tissue specimens, 23 matched pericarcinomatous tissue specimens and 17 normal cervical tissue specimens were obtained from the First Affiliated Hospital of Xi’an Jiaotong University between January 2014 and December 2014. All patients were diagnosed by pathologic examination and none had received chemotherapy or radiotherapy prior to surgery. Every patient was voluntary for this research study and provided written informed consent.

The cervical cancer samples were collected from the center of the tumors, while the samples of pericarcinomatous tissues originated 1.0 cm away from the cancerous tissue. Seventeen samples of normal cervix were obtained from the uterine cervix of patients who underwent a total hysterectomy due to benign uterine diseases. The average size of each sample was 0.5 cm×0.5 cm×0.5 cm. After the tissues were dissected, each sample was washed with sterilized PBS and stored at -80°C. All procedures were performed on ice.

### DNA extraction, bisulfite modification and methylation-specific PCR (MSP)

Genomic DNA was isolated from cells and tissues using a TaKaRa Mini BEST Universal Genomic DNA Extraction Kit (TaKaRa, China) according to the manufacturer’s instructions. A total of 500 ng of the extracted DNA was bisulfite-modified with the EZ DNA Methylation-Gold™ Kit (Zymo Research, USA). The primer pairs used in the MSP were as follows: *SALL3*: 5’-ATTTTAGAATGGAAGGGAGTTCGTC-3’ (forward), 5’-ATAACCTCCTAAAACTTCCCCGAA-3’ (reverse) for methylation and 5’-ATTTTAGAATGGAAGGGAGTTTGTTG-3’ (forward), 5’-AAATAACCTCCTAAAACTTCCCCAAA-3’ (reverse) for unmethylation. Both of the PCR products were 197 bp. The thermocycler programs were as follows: 95°C for 10 minutes and 40 cycles of 95°C for 30 seconds, the annealing temperature for the methylated primer pairs was 51°C while that for the unmethylated primer pairs was 50°C for 30 seconds, and 72°C for 30 seconds followed by an incubation at 72°C for 7 minutes.The PCR products were separated on a 2% agarose gel, stained with Gelview and visualized under ultraviolet illumination. Each reaction was performed in triplicate.

### HPV-DNA testing

HPV-DNA of twenty-three cervical cancer tissue samples and seventeen normal cervix tissue samples were tested by polymerase chain reaction(PCR) and flow-through hybridization. 21 HPV genotypes were qualitatively tested by HPV genotyping test kit according to alkaline phosphatase system(HybriBio,China),including high-risk types HPV-16,18,31,33,35,39,45,51,52,56,58,59 and 68,low-risk types HPV-6,11,42,43,44 and common types in China HPV-53,66 and CP8304.

### RNA extraction and real-time quantitative PCR(Q-PCR)

Total RNA was extracted from cells and tissues using TRIzol Reagent (Life Technologies, USA) according to the manufacturer’s instructions. The primers used for real-time quantitative PCR are as follows: *SALL3*: 5’-CAAAGCGAGCTCAGAAACAG-3’ (forward), 5’-CCTGATGCTCCAACTTCAAA-3’ (reverse); *GAPDH*: *5’*-GCACCGTCAAGGCTGAGAAC-3’ (forward), 5’-TGGTGAAGACGCCAGTGGA-3’ (reverse). The reaction conditions were as follows: 95°C for 30 seconds, 40 cycles of 95°C for 5 seconds and 60°C for 30 seconds. We used the cycle threshold (CT) as the representative point. The relative expression of *SALL3* mRNA in each group (fold-change compared with control) was calculated using the formula: RQ = 2^-△△Ct^. Each reaction was performed in triplicate.

### Statistical Analysis

All the data were analyzed with SPSS version 18.0.Consecutive data were analyzed by the Wilcoxon rank sum test, Kruskal-Wallis H test or one-way ANOVA analysis. Categorical data were compared by Pearson Chi-Square test. All statistical tests were 2-sided, and differences where *p*<0.05 were considered statistically significant.

## Results

### Detection of the methylation status in the promoter regions of *SALL3* in cervical cancer cell lines

**Fig 1:**
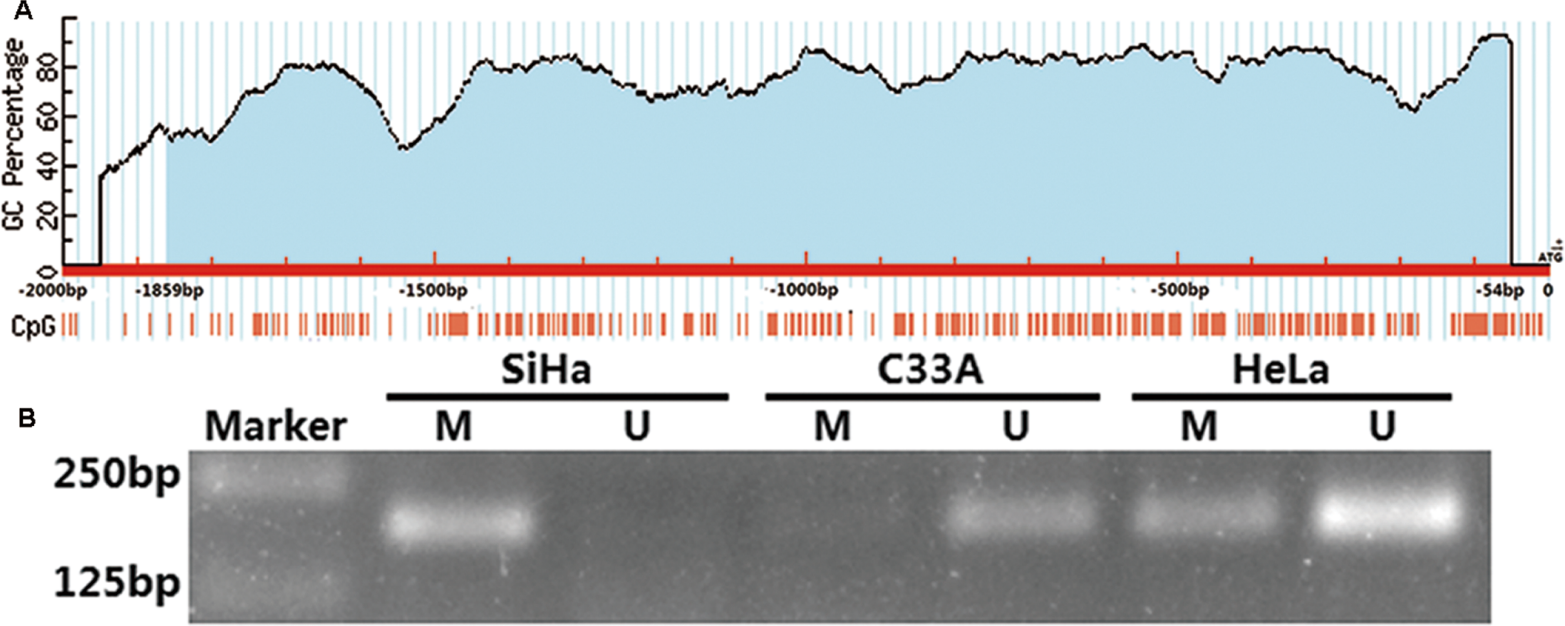
Detection of the methylation status in the promoter regions of *SALL3* in cervical cancer cell lines. (A) Predicted CpG islands in the promoter region of *SALL3*. Numbers indicate the positions in bp relative to the transcription start site. The blue region represents the CpG islands and the red vertical bars are the CpG loci in these input sequences. (B) Detection of *SALL3* methylation status by MSP in SiHa, C33A and HeLa cell lines. (M: methylated; U: unmethylated).

DNA methylation always occurs in areas that are rich in CpG islands such as promoter regions. A common cause of many malignancies is abnormal hypermethylation or hypomethylation of promoter CpG islands, which results in the transcriptional repression of many tumor suppressor genes or the reactivation of oncogenes. We used the online software “MethPrimer” to profile one CpG island in the region that is located from -1859 to -54 bp upstream from ATG, the transcription start site (TSS) in the *SALL3* promoter (Fig 1A). One pair of primers was designed to amplify the *SALL3* promoter region. To identify the methylation status of this region in cervical cancer cell lines, we used methylation-specific PCR (MSP) to determine the *SALL3* promoter methylation status in SiHa, C33A and HeLa cells. The results showed that this region in SiHa cells was completely methylated, whereas this region was completely unmethylated in C33A and partially methylated in HeLa cells (Fig 1B).

### 5-Azacytidine treatment demethylates *SALL3* promoter region and reverses the expression of *SALL3*

**Fig 2:**
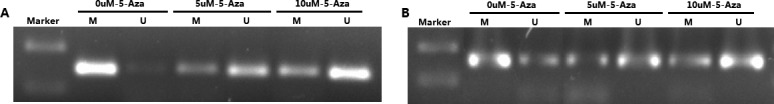
Detection of the methylation status in the promoter regions of *SALL3* in SiHa(A) and HeLa(B) cells after treated with 5-Aza. (M: methylated; U: unmethylated).

**Fig 3:**
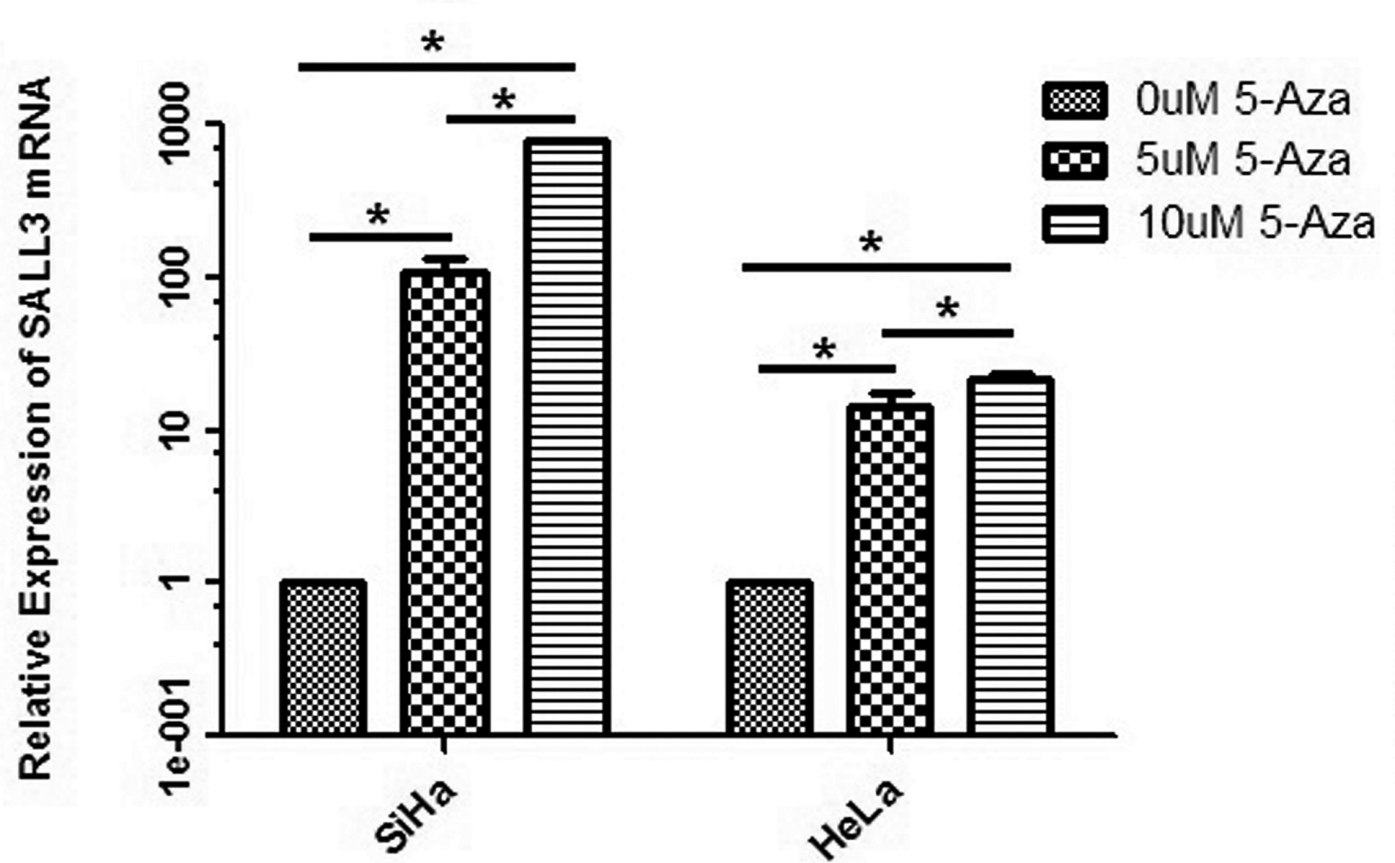
Relative expression of *SALL3* mRNA in SiHa and HeLa cells after treatment with different concentrations of 5-Aza. (*p<0.05).

In most cases, DNA methylation is a reversible process. To identify whether the methylation status in the promoter region regulates the expression of our gene of interest at the transcription level, we detected the expression of *SALL3* in cell lines in which the *SALL3* promoter is strongly methylated (SiHa and HeLa) after treatment with 5-Azacytidine (5-Aza), which can cause DNA demethylation. We measured the methylation by MSP and real-time Q-PCR. The results showed that the intensity of methylation of the *SALL3* promoter in both SiHa and HeLa cell lines decreased, while the unmethylation of *SALL3* gradually increased as the concentration of 5-Aza increased (Fig 2). Moreover, we found that the expression of *SALL3* mRNA in SiHa and HeLa cells increased significantly in a dose-dependent manner after the administration of 5-Aza (*p*<0.05); the increased expression that was observed in SiHa cells was more remarkable than that observed in HeLa cells (Table 1, Fig 3). These results suggested that the expression of *SALL3* was reversed by 5-Aza, which demethylated the *SALL3* promoter and promoted the expression of the *SALL3* gene at the transcriptional level.

**Table 1:** Relative expression of *SALL3* mRNA in SiHa and HeLa cells treated with 5-Azacytidine (5-Aza).

Group	SiHa		HeLa	
	Mean±SEM	*p* value	Mean±SEM	*p* value
0μM-5-Aza	1.00±0.00	^a^0.004*	1.00±0.00	^a^0.002*
5μM-5-Aza	108.60±24.29	^b^0.000*	14.46±2.85	^b^0.035*
10μM-5-Aza	771.43±0.42	^c^0.000*	21.63±1.56	^c^0.000*
Total	---	0.000*	---	0.001*

a. 0 μM compared with 5 μM

b. 5 μM compared with 10 μM

c. 0 μM compared with 10 μM

*statistically significant

### Demethylation of the *SALL3* promoter could inhibit the proliferation of cervical cancer cells

**Fig 4:**
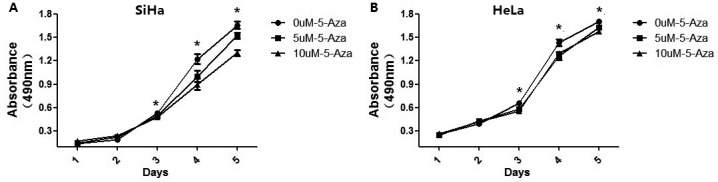
Cell viability and proliferation capacity of the cervical cancer cell lines SiHa(A) and HeLa(B) after treatment with 5-Aza(*p<0.05).

We used an MTT assay to explore whether the proliferation capacity of SiHa and HeLa cells was effected by *SALL3* methylation. After treatment with 5-Aza, the cell viability of both SiHa and HeLa cells was weakened in a dose-dependent manner (*p*<0.05)(Fig 4), which demonstrated that demethylation of the *SALL3* in promoter region could enhance the expression of *SALL3* and lead to a decrease in the cell viability and proliferation capacity of the cervical cancer cell lines SiHa and HeLa.

### The *SALL3* promoter region was hypermethylated in cervical cancer tissues

**Fig 5:**
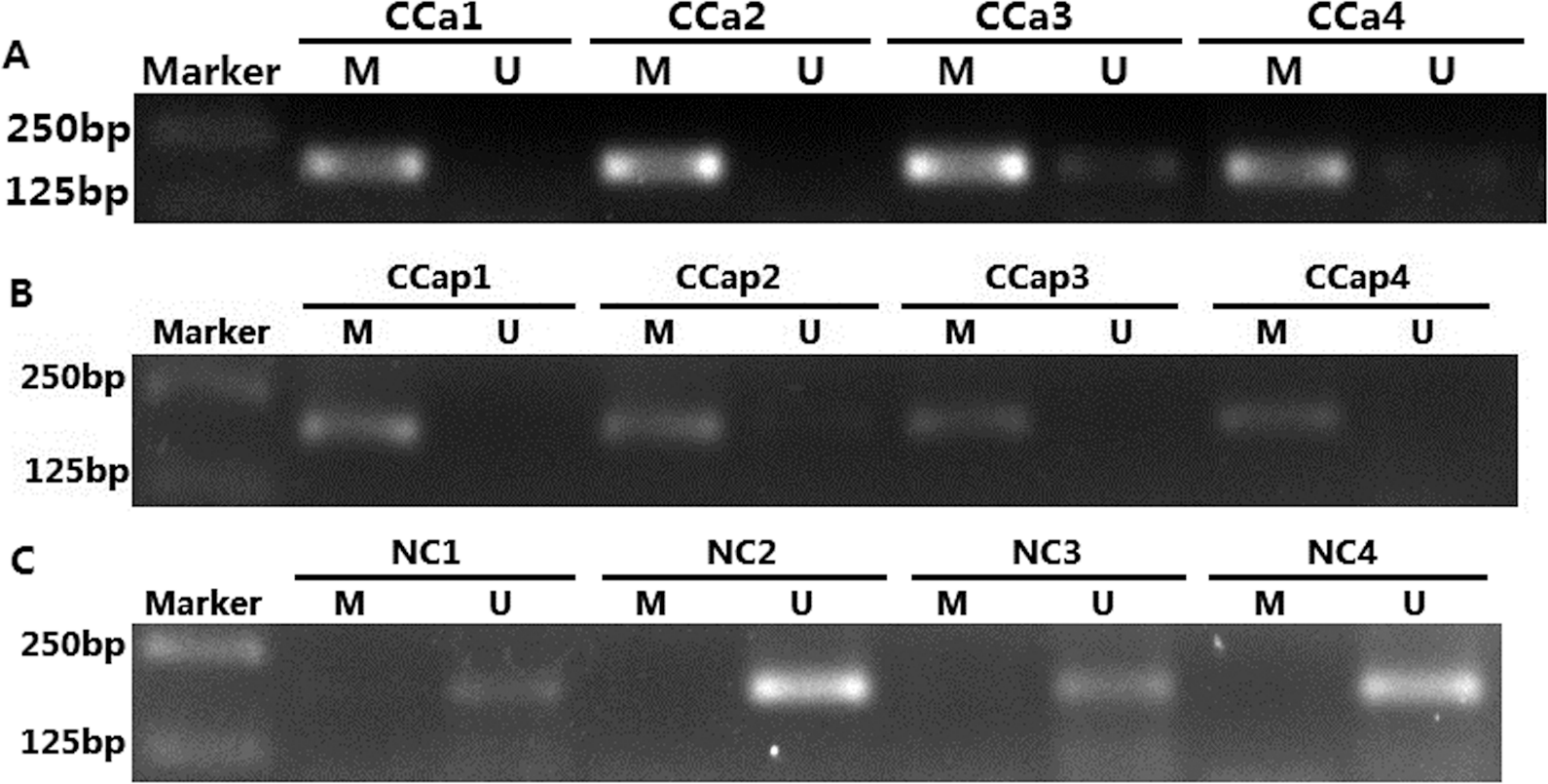
Detection of *SALL3* methylation status by MSP in cervical cancer tissues (A), matched-pericarcinomatous tissues (B) and normal cervix tissues(C) (M: methylated; U: unmethylated).

To further confirm the methylation status of the promoter region of *SALL3* in cervical cancer tissues, we performed MSP in 23 cervical cancer tissues, 23 matched pericarcinomatous tissues and 17 normal cervical tissues. As shown in Table 2,in cervical cancer tissues, 15 were methylated (65.21%) and 8 were unmethylated (34.78); in pericarcinomatous tissues, 15 were methylated (65.21%) and 8 were unmethylated (34.78%); in normal cervical tissues, 5 were methylated (29.41%) and 12 were unmethylated (70.59%). The difference between the groups was significant (*p*<0.05) (Table 2) except that no significant difference was observed between the cancer and pericarcinomatous tissues (p>0.05). However, the average gray-scale level was higher in the cancerous tissues (data not shown). These results demonstrated that the *SALL3* promoter was hypermethylated in cancer tissues, which might play a role in the carcinogenesis of cervical cancer (Fig 5).

**Table 2:** Methylation status of *SALL3* in cervical cancer, pericarcinomatous tissues and normal cervix.

Groups	Methylation (%)	Unmethylation (%)	*p* value
Cervical cancer	15(65.21)	8(34.78)	---
Pericarcinomatous	15(65.21)	8(34.78)	---
Normal cervix	5(29.41)	12(70.59)	---
Total	35	28	0.040*

*statistically significant

### Hypermethylation of the *SALL3* promoter inhibited gene expression at the transcriptional level

**Fig 6:**
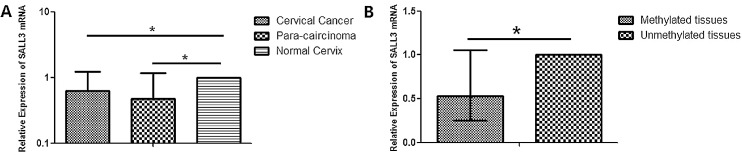
(A) Relative expression of *SALL3* mRNA in cervical cancer, pericarcinomatous and normal cervical tissues. These data are represented by the median with an interquartile range (*p<0.05) (B) Relative expression of *SALL3* mRNA in methylated and unmethylated cervical tissues. These data are represented by the median with an interquartile range (*p<0.05).

We detected the expression of *SALL3* mRNA in the 23 cervical cancer tissues, 23 matched pericarcinomatous tissues and 17 normal cervical tissues by real-time Q-PCR. Compared with the normal cervix, the average relative expression of *SALL3* was lower in cervical cancer tissues (*p*<0.05) and pericarcinomatous tissues (*p*<0.01), but no significant difference was observed between the tumor tissues and pericarcinomatous tissues (*p*>0.05) (Fig 6A).

In addition, we separated these samples into two groups. One group consisted of the tissues where the promoter region of *SALL3* was methylated, and the other consisted of the tissues where that region was unmethylated. The average relative expression of *SALL3* mRNA in the methylated tissues was lower than that in the unmethylated tissues (*p*<0.05) (Fig 6B). These results demonstrated that hypermethylation of *SALL3* inhibited *SALL3* gene expression at the transcriptional level.

### High-risk HPV infection was positively associated with hypermethylation of the *SALL3* promoter region in cervical cancer

Given that high-risk HPV infection is involved in the etiology of cervical cancer, we further explored the relationship between HPV infection and hypermethylation of the *SALL3* promoter region in cervical cancer. In cervical cancer tissues, 14 of 18 HPV-positive samples were methylated (77.78%), and 4 were unmethylated (22.22%); 1 of 5 HPV-negative samples was methylated (20.00%), and 4 were unmethylated (80.00%). In normal cervix tissues, 2 of 6 HPV-positive samples were methylated (33.33%), and 4 were unmethylated (66.67%); 3 of 11 HPV-negative samples was methylated (27.27%), and 8 were unmethylated (72.73%). The combined statistical results were shown in Table 3. The difference between these two groups was significant (*p*<0.05). In addition, we found a positive relationship between HPV infection and hypermethylation of the *SALL3* promoter region in cervix tissues (p = 0.010, r = 0.408)(Table 3), which demonstrated that hr-HPV infection has a close relevance with methylation in the *SALL3* promoter regions.

**Table 3:** The relationship between HPV infection and hypermethylation of *SALL3* in cervical cancer.

Groups	Methylation (%)	Unmethylation (%)	*p* value	*r*
HPV (+)	16(66.67)	8(33.33)	---	---
HPV (-)	4(25.00)	12(75.00)	---	---
Total	20	20	0.010*	0.408

*statistically significant

**Fig 7:**
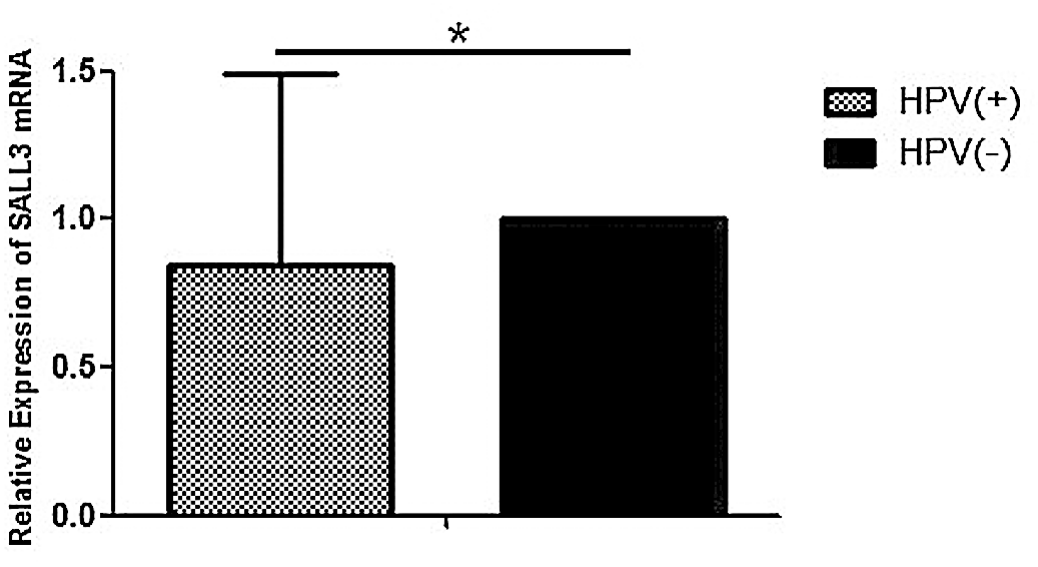
Relative expression of *SALL3* mRNA in HPV-positive and HPV-negative tissues. These data are represented by the median with an interquartile range (*p<0.05).

Moreover, the relative expression of *SALL3* mRNA in the HPV-positive tissues was lower than that in the HPV-negative tissues (Fig 7). The results of HPV types of 40 cervix tissue samples were shown in S1 Table.

## Discussion

On a global scale, the incidence rate of cervical cancer accounts for 13% of all malignant tumors in females and is second only to breast cancer[14, 15]. Although genital HPV has been found to have a close association with the etiology of cervical cancer, and despite the widespread use of screening methods and vaccines[16], the incidence and mortality rate are still high, especially in less developed countries[17–19]. Hence, elucidation of the pathogenesis of cervical cancer is of great importance.

DNA methylation is the most common form of epigenetic modification and is essential for various developmental processes through its regulation of gene expression, genomic imprinting, and epigenetic inheritance[20]. Recent studies have shown that aberrant DNA methylation in promoter regions (and CG islands in particular) is closely related to the genesis of many cancers because a reduction in the methylation of the entire genome or the hypermethylation of promoter regions of tumor suppressor genes affects gene expression[21, 22]. As a consequence, the abnormal methylation of tumor-related genes plays a significant role in carcinogenesis[23]. The discovery of specific methylation markers of different cancers could have a profound impact.

*SALL3* is a member of SAL family locates at 18q23[24], an essential gene in the development of human body. Deletion of 18q23 caused loss of audition, heart problem, mental retardation, growth retardation, limb deformity and so on[7, 24].For the past few years, scientists aimed at the relevance between *SALL3* expression and carcinogenesis. Yu *et al*[25]had found *SALL3* hypermethylated in promoter regions rich in CG dinucleotide in bladder cancer cell lines and tissues, which could be a novel DNA methylation marker in the detection of bladder cancer. What’s more, the promoters of *SALL3* were hypermethylated in H719 cell line[26]. In addition, Yang *et al*[27]discovered that the hypermethylation of *SALL3* contributed to the decrease of *SALL3* mRNA in HCC.

In our study, we have explored the feasibility of detecting hypermethylated *SALL3* in promoter regions as a screening method for cervical cancer. In cervical cancer cell lines SiHa and HeLa, of which were both HPV positive cell lines, were methylated in *SALL3* promoter regions but in C33A, an HPV negative cell line, *SALL3* was unmethylated in the promoter region. Meanwhile, in cervical cancer tissues, matched pericarcinomatous tissues and normal cervix tissues, the results showed hypermethylation of *SALL3* in cervical cancer and pericarcinomatous tissues compared with in normal cervix tissues (*p*<0.05).Our result was consistent with Shikauchi *et al*[8] and Yang *et al*[27].

Taken these together, the infection of HPV might participate in the mechanism of *SALL3* methylation-related carcinogenesis. High-risk HPV (hrHPV)-induced immortalization and malignant transformation are accompanied by DNA methylation of host genes. Schütze *et al*[28]found that HPV-induced immortalization was associated with a sequential and progressive increase in promoter methylation of a subset of genes, including hTERT, mir124–2, PRDM14 and so on, which was mostly independent of the viral immortalization capacity. In our study, surprisingly, by means of analyzing the relationship between HPV infection and *SALL3* methylation status in cervix tissues, the results demonstrated that genomic hypermethylation and lower mRNA expression in transcription level of *SALL3* in HPV-positive cervical cancer tissues when compared with in HPV-negative tissues(*p*<0.05) and hr-HPV infection positively associated with hypermethylation of *SALL3* promoter region (*p*<0.05,r = 0.408),which was consistent with Schütze and our previous study on cervical cancer cell lines, bringing us new evidence that HPV infection did have participated in the carcinogenesis of cervical cancer. However in our present study, the order of two events—hr-HPV infection and *SALL3* methylation occurred and whether they had direct interaction (with E6/E7 or other gene loci) still remains unknown. We’ll keep on searching for the mechanism of carcinogenesis induced by HPV infection and DNA methylation in the following researches.

In order to further verify the relationship between methylation and gene expression, we treated SiHa and HeLa, which were strongly *SALL3* methylated in promoter region, with 5-Azacytidine,an agent could play a role on DNA demethylation on a genome-wide scale. The results suggested that when the concentration of 5-Aza elevated generally, the level of methylation of *SALL3* dropped off as well as unmethylated expression increased significantly in SiHa and HeLa(*p*<0.05). At the same time, the mRNA relative expression of *SALL3* in SiHa and HeLa increase also and the increased extent in *SALL3* totally methylated cell line SiHa was more remarkable than in HeLa, the *SALL3* partial-methylated cell line, which suggested that demethylation of *SALL3* could effectively reverse its expression in transcription level. Then, MTT assay showed that after treated with 5-Aza, the cell viability and proliferation of SiHa and HeLa significantly weakened which illustrated a cancer-inhibiting function of *SALL3* in cervical cancer cells. In tissue samples, the Q-PCR results suggested that mRNA expression of *SALL3* was higher in cancer and pericarcinomatous tissues than in normal cervix tissues(*p*<0.05), which shared the similar idea with previous studies[27]. In addition, statistical analysis of mRNA expression between methylated and unmethylated tissues revealed that in *SALL3* methylated tissues, the mRNA of *SALL3* was higher than in unmethylated tissues(*p*<0.05).Taken together, the results demonstrated *SALL3* hypermethylation contributed to the down-regulation of gene expression in transcription level in cervical cancer.

In conclusion, our research first demonstrated that the promoter of *SALL3* was of hypermethylation, and due to this mechanism down-regulated the mRNA expression and accelerated cell growth in cervical cancer tissues and cell lines. High-risk HPV infection as an etiology of cervical cancer participated in *SALL3* methylation. This aberrant methylation status on the whole genome scale inactivated its function as a tumor suppressor and contributed to the carcinogenesis of cervical cancer.

## Supporting Information

S1 Table HPV types of 40 cervix tissue samples. (DOCX)
