# Supplementary material for: Aberrant Hypermethylation of SALL3 with HPV Involvement Contributes to the Carcinogenesis of Cervical Cancer
Source: PLoS One. 2015 Dec 23;10(12):e0145700. doi: 10.1371/journal.pone.0145700 (PMC4689451; doi:10.1371/journal.pone.0145700)
Supplement: S1 Table — (DOCX) [file pone.0145700.s001.docx]

S1 Table HPV types of 40 cervix tissue samples

|  | Samples | HPV-type |
| --- | --- | --- |
| Cervical Cancer Tissues | Sample 1 | 16 |
|  | Sample 2 | 16 |
|  | Sample 3 | 16 |
|  | Sample 4 | 16 |
|  | Sample 5 | 16 |
|  | Sample 6 | 16 |
|  | Sample 7 | 16,52 |
|  | Sample 8 | 16,33,39,58 |
|  | Sample 9 | 16 |
|  | Sample 10 | 16,33 |
|  | Sample 11 | 16,33 |
|  | Sample 12 | Negative |
|  | Sample 13 | Negative |
|  | Sample 14 | 16,58 |
|  | Sample 15 | 16 |
|  | Sample 16 | 16,58 |
|  | Sample 17 | 16 |
|  | Sample 18 | 16 |
|  | Sample 19 | Negative |
|  | Sample 20 | Negative |
|  | Sample 21 | Negative |
|  | Sample 22 | 16 |
|  | Sample 23 | 16 |
| Normal Cervix Tissues | Sample 1 | 16 |
|  | Sample 2 | 16 |
|  | Sample 3 | Negative |
|  | Sample 4 | 16 |
|  | Sample 5 | 16 |
|  | Sample 6 | Negative |
|  | Sample 7 | Negative |
|  | Sample 8 | 16 |
|  | Sample 9 | Negative |
|  | Sample 10 | Negative |
|  | Sample 11 | Negative |
|  | Sample 12 | Negative |
|  | Sample 13 | Negative |
|  | Sample 14 | Negative |
|  | Sample 15 | Negative |
|  | Sample 16 | 16 |
|  | Sample 17 | Negative |
